# Efficient Approach for Anomaly Detection in IoT Using System Calls

**DOI:** 10.3390/s23020652

**Published:** 2023-01-06

**Authors:** Nouman Shamim, Muhammad Asim, Thar Baker, Ali Ismail Awad

**Affiliations:** 1 Department of Computer Science, National University of Computer and Emerging Sciences, Islamabad 44000, Pakistan; 2 School of Architecture, Technology and Engineering, The University of Brighton, Brighton BN2 4GJ, UK; 3 College of Information Technology, United Arab Emirates University, Al Ain P.O. Box 17551, United Arab Emirates; 4 Centre for Security, Communications and Network Research, University of Plymouth, Plymouth PL4 8AA, UK

**Keywords:** Internet of Things, security, anomaly detection, system calls, dynamic threshold

## Abstract

The Internet of Things (IoT) has shown rapid growth and wide adoption in recent years. However, IoT devices are not designed to address modern security challenges. The weak security of these devices has been exploited by malicious actors and has led to several serious cyber-attacks. In this context, anomaly detection approaches are considered very effective owing to their ability to detect existing and novel attacks while requiring data only from normal execution. Because of the limited resources of IoT devices, conventional security solutions are not feasible. This emphasizes the need to develop new approaches that are specifically tailored to IoT devices. In this study, we propose a host-based anomaly detection approach that uses system call data and a Markov chain to represent normal behavior. This approach addresses the challenges that existing approaches face in this area, mainly the segmentation of the syscall trace into suitable smaller units and the use of a fixed threshold to differentiate between normal and malicious syscall sequences. Our proposed approach provides a mechanism for segmenting syscall traces into the program’s execution paths and dynamically determines the threshold for anomaly detection. The proposed approach was evaluated against various attacks using two well-known public datasets provided by the University of New South Mexico (UNM) and one custom dataset (PiData) developed in the laboratory. We also compared the performance and characteristics of our proposed approach with those of recently published related work. The proposed approach has a very low false positive rate (0.86%), high accuracy (100%), and a high $F_{1}$ score (100%) that is, a combined performance measure of precision and recall.

## 1. Introduction

The Internet of Things (IoT) is a vast network of smart devices or “things” capable of exchanging data with other devices and systems over the internet. From its very outset, the IoT has been envisioned as an integration of people, technology, and processes for remote monitoring, evaluation, and manipulation of systems. Over time, it has evolved with progress in related technologies such as wireless communication and ubiquitous computing. At present, the IoT is a confluence of 5G, cloud computing, machine learning, and artificial intelligence, and has offered improved and cost-effective ways to control, monitor, and evaluate systems [1]. This potential of IoT is quickly realized and leads to the rapid growth and adoption of smart devices in conventional systems. The field found many innovative and futuristic use cases, such as Industry 4.0 [2], the Internet of Medical Things [3,4], smart cities [5], smart transportation systems [6], and predictive maintenance [7]. IoT is evolving and is expanding at a fast pace, currently, there are around 14 billion IoT devices globally [8,9,10]; this number is expected to reach 30 billion [11] by the year 2025. The situation indicates that IoT is an important and effective technology having a key role in modern and future systems.

As a disruptive technology, the IoT is swiftly adopted at a large scale before the testing, evaluation, and quality assurance standards can be in place. This has resulted in a large number of IoT devices being installed with a number of potentially obscure software and hardware flaws, such as weak security mechanisms, vulnerable application programming interfaces, and logical programming errors [12]. Consequences of these flaws and weaknesses are realized as malicious actors using these devices as entry points to connected systems, as part of larger attacks. There have been incidents where an insecure IoT device acted as a weak link in the chain of a relatively secure setup and compromised its security, such as a fish-tank attack [13]. In most of these attacks, commodity devices, such as smart cameras, printers, fax machines, and digital locks have been exploited [14,15]. However, in some cases, industrial systems of critical nature have been successfully attacked, resulting in significant impacts [16]. Another serious security concern related to IoT devices is that malicious actors can form large botnets from these devices and launch a large-scale distributed attack, such as the Mirai botnet attack [17,18]. This has raised serious concerns regarding the security and privacy of IoT-enabled systems and requires effective countermeasures.

To safeguard smart devices against cyber threats, IoT security has been actively researched from various perspectives and various approaches have been proposed [19,20]. The developed approaches can be broadly grouped into two categories: signature-based and anomaly-based approaches [21,22,23]. Signature-based approaches use the characteristics of existing attacks to build detection models that are typically robust against known attacks. However, they lack the ability to handle novel attacks and must be updated consistently. Anomaly detection approaches model the normal behavior of the target system and detect deviations from it; these deviations are considered anomalies or malicious activities. These approaches have some advantages over signature-based approaches, as they can detect novel and unknown attacks and require only normal data for training. Anomaly detection approaches, however, have their own limitations; the chief among them is learning all benign behaviors and a high false-positive rate.

The efficacy and efficiency of an anomaly detection approach are largely influenced by the data used to represent the system’s normalcy; for example, using network traffic data will affect anomaly detection accuracy owing to inherent communication-related problems. Several anomaly detection approaches have demonstrated that a device’s system call (syscall) data can be very effective for attack and anomaly detection. Syscall data are ordered sequences of system calls from one or more processes acquired during execution using special tools, such as ptrace and strace [24]. The data must be broken down into smaller units before being passed to the anomaly detection system to learn normal behavior and anomaly detection. The length of these smaller units (referred to as segments) plays an important role in representing the system features and consequently affects its performance. For example, the well-known *n-gram* [25] approach is usually employed in language-model-based anomaly detection approaches for feature representation. Here, the value of *n* represents the number of words or syscalls to be treated as a training example, which plays a critical role in the performance of the model. A very small value of *n*, such as 1, does not capture any useful patterns, whereas a large value of *n* increases the space complexity of the model by many folds.

Anomaly detection approaches usually measure the deviation from normal behavior and require a way to determine how much deviation should be considered an anomaly. Considering a slight change in normal behavior as an anomaly will lead to a high false positive rate, whereas allowing too much deviation will result in poor detection of anomalies. A common approach to handle these issues is to determine suitable parameter values through trials and tests, such as a fixed threshold or segment length, in our case. However, this makes the model specific to the system and data, and cannot be generalized. A better method is to dynamically determine the parameter values according to the underlying system and data so that the model can be generalized.

To address these issues, we propose a simple and lightweight anomaly detection approach that dynamically determines the threshold for anomaly detection and present a mechanism for segmenting syscall sequences. The proposed approach considers syscall data as a long sequence of program iterations and attempts to estimate and extract individual iterations. The execution of the underlying program is modeled as a Markov chain, and the iterations are considered paths in this chain. We used the probabilities of individual iterations or paths to learning the thresholds for anomaly detection. The approach was devised considering the following assumptions: (a) the host application in an IoT device is active all the time, and (b) all execution paths of a program have some degree of similarity, which are fundamental features of common IoT devices.

The main contributions of the proposed approach are summarized as follows.

1. We propose a new method to dynamically segment the syscall data into constituent program execution paths. The method is based on the novel idea of treating the syscall sequence as a sequence of program execution paths (Section 4). It finds a start-of-execution pattern for these execution paths and segments the syscall sequence using this pattern. Hence, it presents a solution to the issue of using a fixed segment length, which makes model data specific. Related discussions are in Section 2.1, Section 3.1 and Section 4.1.
2. We propose a novel method to address the issue of using a predetermined threshold in probabilistic anomaly detection approaches for the IoT (Section 4). The devised method is based on the idea of presenting syscall segments as paths of a Markov chain. The threshold is dynamically determined using the probabilities and lengths of these paths, Section 2.1 and Section 3.2 provide the related discussion.
3. An anomaly detection approach for IoT devices that is computationally efficient and that has accuracy and false positive rates better than existing approaches (Section 4 and Section 6).
4. We propose a syscall dataset that consists of long traces of normal activities of a custom IoT device captured under a variety of standard conditions along with multiple attack traces (Section 5.1.2). There are only a few syscall datasets available in this domain, and the proposed dataset can be helpful for further research in this area.

The remainder of this paper is divided into the following sections: Section 2 covers related work and highlights related challenges in the IoT anomaly detection domain, and discusses their relevance to the proposed approach. Section 3 provides related background knowledge. Section 4 presents a conceptual design of the proposed approach. Section 5 describes the testing and evaluation of the proposed approach. Section 6 presents the results of the testing and evaluation processes, along with performance comparisons and a discussion. Finally, the concluding remarks and future work are presented in Section 7.

## 2. Related Work

Anomaly detection is considered an effective approach for detecting attacks and malicious activity in IoT devices. Research in this area has considered various tools and techniques from various domains, such as machine learning, statistics, probability, model checking, rule checking, deep learning, and finite state automaton. Popular data sources used in existing approaches include network traffic, syscalls, program code, and, in some cases, device utilization data. This section discusses various aspects of the existing IoT anomaly detection approaches that have some degree of relevance to the proposed approach.

We first discuss anomaly detection approaches that follow the security models devised by conventional networks. Most of these approaches utilize IoT network traffic data to learn normal behaviors or benign communication patterns. A good example is the work of Sivanathan et al. [26], which is based on the network flows of IoT devices under normal and attack conditions. An extension of this study is [27], which uses packet-level network traffic data for the same objective. In these approaches, anomaly detection is accomplished using a one-class support vector machine (SVM) and K-means clustering. The work of Eskandari et al. [28] is also based on the network flows of IoT devices, where two standard machine learning models, i.e., isolation forest (IF) and local outlier factor (LOF), are used for anomaly detection. Similarly, Maniriho et al. [29] used machine learning, i.e., random forest (RF), on IoT network traffic for anomaly-based attack detection, analyzed the relatedness of data features using information gain and set theory, and proposed a hybrid feature selection engine.

Network traffic-based anomaly detection falls into the category of network-based intrusion detection systems and is deployed in a central entity, typically a router or an IoT gateway. Commonly associated challenges with these approaches are the single point of failure and the inability to scale well and detect novel attacks. In addition, many other factors are difficult to avoid and can lead to a high false-positive rate. For example, traffic noise, network congestion, device failure, and changes in network conditions. Approaches that handle some of these challenges simultaneously, such as [29] provide a method for handling changes in network conditions. These challenges are better addressed by distributed, collaborative, and host-based anomaly detection approaches.

Mirsky et al. [30] is an example of a collaborative anomaly detection approach for IoT. Participating IoT devices first learn a local anomaly detection model, and then share it over a blockchain to produce a single agreed-upon version. This final version is used by IoT devices to detect anomalies locally. The approach uses the jump sequence of the program instruction and a Markov chain to model normal behavior; for anomaly detection, a minimum probability threshold is used. However, the approach has many limitations, for example, the threshold is fixed and data-specific, and participating devices need to have specific hardware architecture and application software.

The lack of heterogeneity in the aforementioned approach was handled well by Nguyen et al. [31]; they proposed a distributed learning approach that first identifies the device model and allows only similar devices to learn collaboratively. In this approach, network traffic data are first transformed to a specific format using a language model and then fed to a gated recurrent unit (GRU) to estimate the probability of data being anomalous. The approach is novel, can scale well, and can be generalized, with limitations on device-type identification, fixed detection, triggering thresholds, and fixed look-back window size.

Many of the approaches discussed above use machine learning models, which are relatively resource-intensive and, in some cases, are not feasible for resource-constrained IoT devices. In view of this, many non-machine learning-based anomaly detection approaches have been proposed. Notable among them is the work of Wang et al. [32], who proposed a novel approach to capturing the normal behavior of IoT devices using device usage rules (DUR). The proposed approach automatically extracts DURs from the manufacturer usage description (MUD) [33] files and device interaction rules from the triggering platform IFTTT [34] used by the devices. The DURs are passed to a rule engine (Drools [35] in this case) for the real-time monitoring of IoT devices. The approach is novel; however, the concept of MUD has limited support from vendors and is not mature. Similar to this is the work of Sharma et al., in their work [36] they proposed a lightweight formal verification method of the device’s operational specification, the developed approach not only captures the normal behavior but also determines a complete set of prohibited states of devices. Despite being lightweight and effective (as claimed), the approach is not scalable and generalizable owing to very specific requirements, such as operational specifications.

Considering anomaly detection approaches based on syscall data, Forrest et al. [37] pioneered the use of system calls for anomaly detection and showed that short sequences of system calls of a process generate a stable signature of its normal behavior. They experimented with various Unix processes and proposed an effective anomaly detection approach. The approach has two major issues: first, the segment length is selected using experimental results, and second, it requires a repository of segments that can lead to high space complexity for long syscall datasets. The authors in [38] adopted the same approach; however, they used a fixed-length sub-sequence, and Eskin et al. [39] improved this work by using a dynamic sub-sequence length and also showed that the length of the sub-sequence impacts the anomaly detection accuracy.

Similar to this is the work of Hoang et al. [40] in which two long short-term memory (LSTM) networks are trained using normal data and attack syscall data. For a given syscall sequence, both models estimate a probability value, and a higher probability is considered the final result. The authors reported an important observation that the length of the syscall sequence directly affects the performance of their model, i.e., their approach performed best for a particular length of the syscall sequence (150 in this case) and performed poorly otherwise. The approach proposed by Liao et al. [41] attempts to find suitably long sub-sequences of syscall data; however, anomaly detection is performed using an SVM. This approach also supports the fact that the sub-sequence length is an important parameter for differentiating between normal and abnormal syscall sequences. Their proposed approach used a mix of fixed and variable length sub-sequences to train an SVM classifier and achieved a very high accuracy with a very low false positive rate. Similarly, [40,42] used an n-gram of syscall with machine learning approaches, specifically LSTM, one-class SVM, and clustering for detecting malicious syscall sequences. These approaches empirically find a suitable length for n-grams to build the model; thus, they face generalization issues.

Statistical feature analysis is also an important area in syscall-based anomaly detection for IoT devices. A common strategy among these approaches is to segment long syscall sequences into short sub-sequences of fixed or variable lengths, extract statistical features of these sub-sequences, and use the extracted features to build an anomaly detection model. The sliding window, n-gram model, and suffix tree are generally used to obtain fixed- and variable-length sub-sequences, respectively. Language models and one-class classification are commonly applied for anomaly detection. Liu et al. [43] proposed a cross-platform anomaly detection model using the statistical features of syscall sequences. A similar cross-platform anomaly detection approach was proposed by Zhang et al. [44], who proposed a framework that first enhances the behavioral semantic information contained in syscall sequences and produces generalized features, which are passed to TextCNN, which is a convolutional neural network (CNN) for text classification. The approach has much better accuracy than that of Zhang et al. [44]; however, it has a high computation cost for training and updating.

Breitenbacher et al. [45] proposed a unique anomaly detection approach that monitored process spawning and allowed only legitimate processes to be executed. The model uses a ’whitelisting’ of the legitimate processes built during the training phase and the real-time anomaly detection model must be loaded as a kernel module. The approach is novel, effective, and efficient, and requires low computational resources; however, whitelisting processes for a variety of IoT devices and embedding the module into the kernel is a challenging task. Similarly, Carter et al. [46] considered that reducing the feature dimension affects the performance of anomaly detection approaches, which is crucial for resource-constrained IoT devices.

Syscall anomaly detection approaches employ a variety of tools and techniques ranging from simple statistical analysis to complex deep learning models. We can summarize the common system-based IoT anomaly detection approaches as (a) statistical feature analysis, (b) language modeling, (c) model checking, (d) formal specification, and (e) sequence analysis. Overall, syscall-based anomaly and attack detection approaches are very effective for IoT devices because of their role in program execution.

### 2.1. Research Gap Analysis

Based on our study of existing approaches, we found that host-based anomaly detection approaches are more effective than network-based approaches and that host-based approaches can be further extended to collaborative or distributed anomaly detection systems. By analyzing existing IoT anomaly detection approaches, we discovered that syscall data can be very effective for anomaly detection in IoT and can provide an excellent trade-off between generalization and accuracy. Table 1 represents a summary of the related studies focusing on the used approaches, methods, and datasets. Regarding the existing syscall-based IoT anomaly detection approaches, we found that these approaches lack (a) an automated and dynamic mechanism for the conversion of long syscall sequences into individual training/test examples, and usually, this is done empirically, and a suitable length is selected for which model performs best; (b) a method to automatically determine the extent to which the system can deviate from normal, usually a threshold is used for this purpose, which is empirically determined. These two issues impose great restrictions on the generalization of anomaly detection approaches in this area.

## 3. Background

This section provides a brief description of the concepts related to system calls and the Markov chain (MC) and discusses the relationship between syscalls and the execution behavior of an IoT device.

### 3.1. System Calls and Program Execution Path

**Figure 1:**
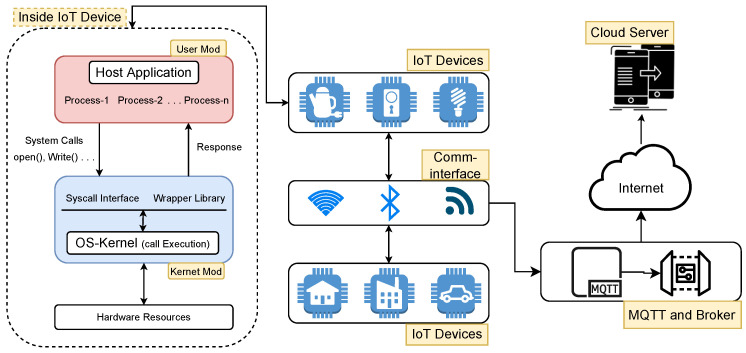
Abstract view of the IoT and system calls (syscalls).

The core of an operating system (OS) consists of a kernel, which is a special program that assists other parts of the OS to perform their tasks. Specifically, it controls and manages access to system resources, such as CPU and disks, and acts as an interface between user programs and OS services. For security and control management, the OS uses two different modes for program execution: the user mode and kernel mode. A program is usually executed in the user mode; however, certain critical operations, such as disk read/write and process creation, can only be executed in the kernel mode. For such operations, the program requests kernel services employing special functions called system calls, which are then carried out by the kernel in kernel or privileged mode. An abstract view of the entire process is shown in Figure 1.

System calls made by a program can reveal important information about its activities. Usually, when a program executes, it makes a number of system calls in a particular sequence, and each execution sequence defines the execution path of the program. There can be only a limited number of execution paths for a program, some of which might occur more frequently than others, such as in the case of the main and optional functionalities of a program.

The execution cycle of a program consists of call sequences from the start of the program to its end; it can be a unique execution path or consist of many constituent execution paths. A program that, once loaded and remains active, usually executes a part of the program repeatedly and does not execute the start and end of the program in each execution cycle. Usually, IoT application programs, once started, will remain alive and execute their main functionality in a repeated manner, such as in the case of temperature-sensing IoT devices. In some cases, the application program can be impetus-based, such as a smart smoke detector, and some functionality of the program will only be executed when the triggering condition is met. Even in such cases, a repeated execution pattern is exhibited owing to the limited functionality of the application program. IoT devices are usually designed for a few specific tasks, and the application programs for these tasks are relatively small and simple. It can be expected that the underlying execution paths will be very few and of short lengths.

The behavior of an application program can be defined in terms of its execution paths, and many existing approaches attempt to accomplish this in various ways, such as defining control-flow graphs [49], building finite-state automata, or formally modeling the application [50]. These approaches utilize the fact that any modification in the program’s execution path is due to the modification in the program’s code or related device conditions, such as the allocation of memory or the creation of a socket. The aforementioned conditions, however, can also be caused by a number of malicious actions, such as code injection, code reuse, buffer overflow, and a denial-of-service attack, or can be due to benign reasons, such as a faulty network. The proposed approach utilizes this characteristic, attempts to identify any change in the program’s execution path, and determines whether the change was due to benign or malicious activity.

### 3.2. Markov Chain

A Markov chain (MC) models the transition probability from a given state to the next state. It is a memoryless process, i.e., transition probability to a state depends only upon the last state and not upon all previous states. More formally, MC is a stochastic process $\left\{X_{0},X_{1},X_{2}\ldots \right\}$ where $X_{t}$ represents the state of the system at time *t* that satisfies the Markov property, i.e., the state $X_{t+1}$ depends only upon the previous state $X_{t}$.

(1) $$P\left(X_{t+1}=s|X_{t}=s_{t}\right)\;\forall t=1,2,3,\ldots \;\mathrm{and}\;\forall \;\mathrm{states}\in S=s_{0},s_{1},s_{2},\ldots s_{t},s$$

where *S* denotes the system state space.

This is also called the first-order Markov chain, the order of the MC is the number of preceding states required for the next state, in an n−degree MC, the next state depends upon *n* previous states. Typically, MC is described by a transition matrix and an n-state MC chain is represented by the nxn transition matrix. Rows in the transition matrix define now or $X_{t}$ while columns represent then or $X_{t+1}$. An entry (i,j) in the transition matrix is the conditional probability (p) of going from state i to state j.

(2) $$p_{ij}=P\left(X_{t+1}=j|X_{t}=i\right)$$

A trajectory in the MC chain is a sequence of states or a path followed by the system. The probability of a trajectory is determined by multiplying the conditional probabilities of the states in the trajectory, i.e., the probability of the trajectory $s_{0},s_{1},s_{2},\ldots ,s_{t}$ is given by

(3) $$P\left(X_{0}=s_{0},X_{1}=s_{1},\ldots ,X_{t}=s_{t}\right)=p_{s_{t-1},s_{t}}\times p_{s_{t-2},s_{t-1}}\times \ldots \times p_{s_{0},s_{1}}\times \pi_{s_{0}}$$

where $\pi_{s_{0}}$ is the initial probability of states $s_{0}$ and π is the initial probability distribution.

A transition matrix is typically constructed by first finding an N × N frequency matrix $N_{i,j}$, where N is the number of system states. For each transition from state i to state j, entries i and j into N are incremented. The transition matrix M and initial probability distribution can then be obtained by the following equations [51,52]:

(4) $$M=\frac{N_{ij}}{N_{i}}$$

(5) $$\pi =\frac{N_{i}}{N}$$

We find MC suitable for our desired framework as follows: (a) constructing M from the syscall sequence is computationally inexpensive and requires a very small amount of memory, (b) the size of M is independent of the length of the syscall sequence, and (c) M can be updated easily by simply incrementing the frequency of encountered states in the frequency matrix and updating the respective probabilities in the transition matrix (4). Our proposed approach is based on the novel idea of using program execution paths as trajectories in the respective MC. We used the probabilities of these trajectories (3) to dynamically determine a threshold for distinguishing between normal and abnormal executions. Syscalls appearing frequently will have a higher probability than less frequent ones, and it is likely that a sequence of normal syscalls will have a higher probability than an attack or anomalous syscall sequence.

One way to capture all possible normal syscalls is to observe the host for a very long time; however, this only reduces the chances that a new normal call will appear during testing. For example, in our case, if normal data consist of more than $10^{6}$ syscalls, the initial probability of a normal call will be $1\times 10^{-6}$, and any normal call having a true probability less than this will not appear in the training data. To address this situation, new syscalls are added to the transition matrix with a very low probability, such as $1\times 10^{-7}$ in the example being discussed, which will have a small effect on the probabilities of normal sequences from (3), and anomalous sequences will still be distinguishable from normal sequences. A normal call will eventually be repeated, and its probability will continue to improve; however, an anomalous call might never repeat, and its probability will remain the lowest. Another way to handle new normal calls is to simply assign them a zero probability, in which case the MC model does not need to be updated, and any of the two approaches can be used depending on the behavior of the host device.

## 4. Proposed Approach

The core idea of our proposed approach is to transform the long syscall sequence into program iterations, determine the probability of each iteration, group these probabilities according to the length of the iteration, define a minimum threshold for each group, and use these thresholds to differentiate between normal and anomalous program executions. For this transformation, we propose a segmentation approach; for probabilities, we use the Markov chain model. This section provides details of these steps, related challenges, and the rationale of the proposed solutions.

**Figure 2:**
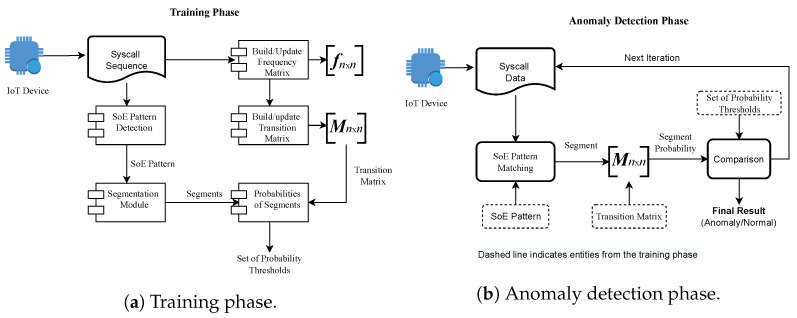
Block diagram of (**a**) training and (**b**) anomaly detection phases of the proposed approach.

There are two phases and three major components of the proposed approach. Figure 2 provides a block-level description of the overall approach. We named the two-phase training and anomaly detection, where the components are (a) segmentation mechanism, (b) transition matrix, and (c) a set of thresholds.

### 4.1. Segmentation Mechanism

**Figure 3:**
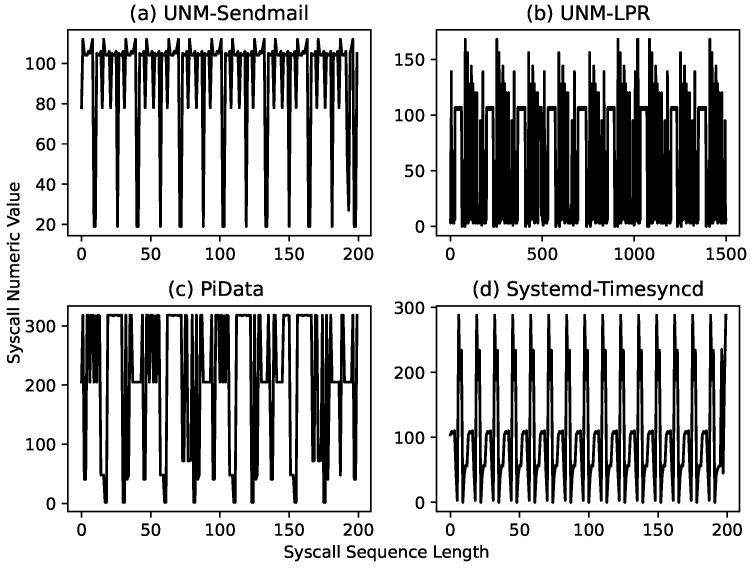
Line plot of syscall sequences taken from three datasets (**a**–**c**) and a standard Linux process (**d**). Each syscall sequence exhibits a repeated pattern of varying length, for example, the UNM Sendmail plot shows that the syscall pattern is repeated after 15 calls, whereas in the UNM-LPR plot, the syscall pattern is repeated after approximately 166 syscalls.

As explained earlier, the syscall sequence is usually a long sequence comprising data from the repeated execution of the program, as shown in Figure 3. We consider these repeating patterns as program iterations; the high degree of similarity among these iterations indicates the presence of syscall subsequences common to all iterations. We take the longest of these subsequences and segment the syscall sequence at the starting position of this subsequence, which we consider as the start of execution (SoE) pattern of an iteration, although it can be any part of it.

To find the SoE pattern, we performed an initial segmentation using the autocorrelation function (ACF) and then obtained the longest common subsequence (LCS) from the resultant segments. The reason for this initial segmentation becomes clear as we proceed with this section.

**Figure 4:**
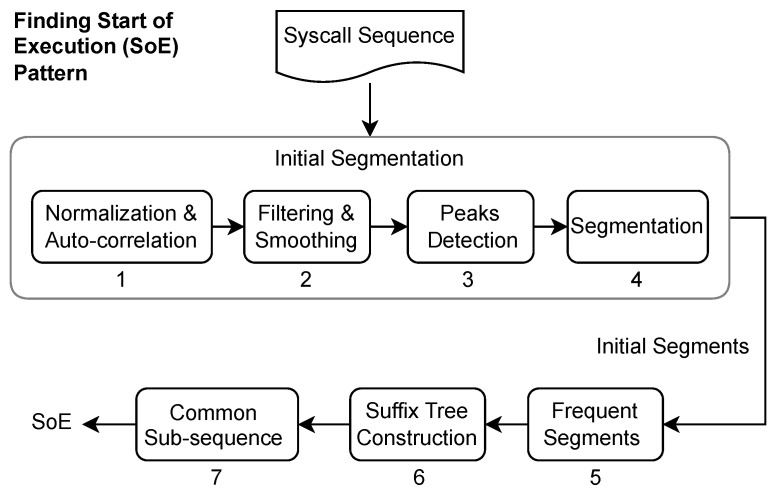
Block diagram showing the process of finding the start of execution pattern: Steps 1 to 4 show the initial segmentation, step 5 uses these initial segments to find the most common segments, step 6 is the construction of the suffix tree, which returns the sub-sequences common among these initial segments.

Autocorrelation is the correlation between a sequence or time series with a delayed or lagged version. The value of correlation is high when the two sequences match, negative for complete mismatch, and varies between partial matches. ACF has the property that autocorrelation of a periodic function is also periodic, which means that if our syscall sequence has a repeated pattern, ACF will produce relatively high values or peaks at regular intervals. We computed these peaks and performed our initial segmentation using the positions of these peaks in the syscall sequence, the entire process of finding the SoE pattern is shown in Figure 4.

**Figure 5:**
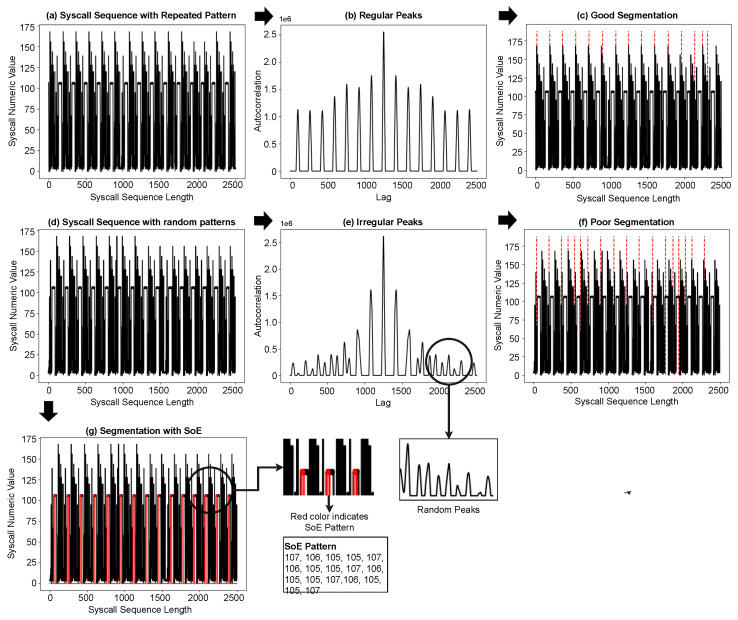
(**a**–**c**) show a sample of the UNM-LPR syscall sequence, its autocorrelation, and segmentation, respectively. The dotted red lines in (**c**,**f**) show the segmentation boundaries determined using positions of peaks from autocorrelation; (**d**–**f**) show the same stages for a noisy UNM-LPR syscall sequence sample, while (**g**) shows boundaries determined using the SoE pattern shown in red; (**g**) shows that the SoE pattern provides better segment boundaries for a noisy syscall sequence for which the autocorrelation method did not perform well.

The initial segmentation described above works perfectly well as the final segmentation if there is only one pattern with perfect repetition. During this initial segmentation, we discovered that (a) there are variations in the periodicity of the syscall sequence, and (b) there can be several random peaks between two large peaks, which leads to poor segmentation. The change in periodicity seems natural, as program execution can be altered by errors, exceptions, and logical conditions. The second issue is caused by a change in periodicity, autocorrelation of a variable length repeating pattern causes many partial matches and results in these random peaks between, which need to be removed or ignored before segmentation. We handled the peak-related challenge by applying the Savitzky Golay smoothing filter [53] with a window size of 31, the said filter is well known for smoothing noisy peaks and sharpening large peaks, Figure 5 shows the results of this smoothing operation.

We grouped these initial segments according to their length, and the group with the largest number of segments was used to find the LCS. A suffix tree was built from the selected group of initial segments to obtain the LCS among these segments. The LCS obtained was considered an SoE pattern and was later used for the final segmentation. At this point, it is important to explain why the initial segmentation is not as good as the final solution and why the suffix tree is built from initial segments instead of the entire syscall sequence. The initial had two main issues (a) it was noisy, (b) the autocorrelation peaks can be caused by partial or perfect match, (c) it is challenging to determine the exact parts of the two sequences that actually matched, Figure 5 shows the results of segmentation using ACF and SoE.

Finding an LCS is a challenging problem, and a trivial solution, such as dynamic programming, would take quadratic time; however, a generalized suffix tree algorithm, specifically Ukkonen’s algorithm [54], which is used here, solves this in θ(n) in the time and space domains, where n is the length of the sequence.

### 4.2. Transition Matrix

As explained in Section 3.2, for each dataset, we first determined the frequency matrix $f_{nxn}$ and then derived a transition matrix $M_{nxn}$ from it. The transition matrix was later used to determine the probabilities of segments during the training and anomaly detection phases. The size of $M_{nxn}$ in each case was small, as the number of unique syscalls in any dataset did not exceed 60.

### 4.3. Set of Thresholds

After segmentation, a Markov chain was used to determine the probabilities of the segments. The probabilities are then grouped according to the length of the segments, and the collection thus formed consists of several small groups, each containing one or more members. For each group, we considered the minimum probability and used it as a threshold for segments with lengths close to this. The final outcome of this step is a dictionary *T* consisting of key-value pairs (l,p) where the key *l* represents the segment length, and the value *p* is the threshold at this length.

### 4.4. Training Phase

Figure 2a provides an overview of the training phase, and the two initial steps, i.e., construction of the Markov chain and discovery of SoE, can be performed in any order after finding the SoE. Segmentation is performed, and the set of thresholds *T* is determined using the transition matrix *M*. To be able to use *M*, we needed it to reach a state where the state transition probabilities reach a fixed value or an equilibrium state. One way to achieve this is to use a large training dataset. To determine whether *M* reached a stable condition, the following equation is used, which simply measures the difference between two consecutive states $S_{i},S_{i+1}$ of *M*.

(6) $$Diff_{i}=S_{i}-S_{i-1}\;0<i\leq n\;(\mathrm{Sequence}\;\mathrm{Length})$$

**Figure 6:**
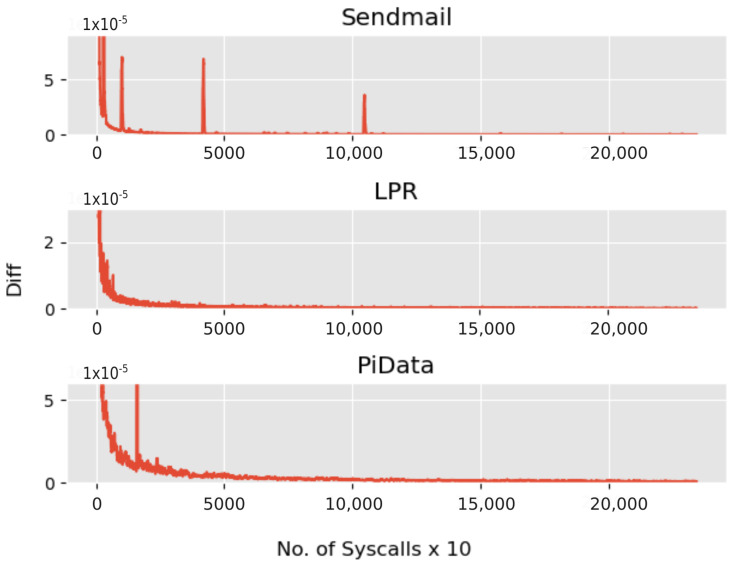
Markov chain convergence.

Figure 6 shows the convergence of *M* for each dataset used, showing that in each case, *M* converged within the training data.

### 4.5. Anomaly Detection

During the anomaly detection phase, the given syscall data are sequentially read and scanned for the SoE pattern. Syscall sequence from the start of an SoE pattern to its next occurrence is extracted and taken as an iteration of program execution, which we call the candidate segment $s_{c}$. The probability $p_{c}$ of each $s_{c}$ is determined using the transition matrix *M* learned during the training phase and the length $l_{c}$ of the candidate segment is used to determine the correct threshold $thresh_{l}$ from the set of thresholds *T* obtained during the training phase. The anomaly detection is then computed as

(7) $$f\left(l_{c},p_{c}\right)=\left\{\begin{array}{cc} 1 & \;\mathrm{if}\;l_{c}\in T\;\mathrm{and}\;p_{c}\geq thresh_{l}\\ 0 & \;\mathrm{if}\;l_{c}\notin T\\ 0 & \;\mathrm{if}\;l_{c}\in T\;\mathrm{and}\;p_{c}<thresh_{l} \end{array}\right.$$

where *f* represents the anomaly detection function, 1 represents a normal segment, and 0 represents an anomaly.

To allow room for small variations in length, we relaxed the length-matching criteria to some percentage of the candidate segment length. If there is no matching group against $l_{c}$, we allowed using a group of lengths up to 5% smaller than $l_{c}$. We allowed a smaller group as the probability of segments decreases with an increase in length, which makes it difficult to meet the probability criteria of a smaller length group.

## 5. Testing and Evaluation

Seven sets of attack traces from two datasets were used to determine the performance of the proposed approach. Several performance metrics were computed for each normal and attack trace, and compared with existing work. This section provides details on the datasets, attacks, performance metrics, and work selected for comparison.

### 5.1. Datasets Characteristics

We used a total of four sets of syscall traces, three of which are from the UNM public dataset [55] and one is our custom dataset, which we named PiData. First, we discuss the UNM dataset and provide a description of PiData. A summary of these datasets is provided in Table 2.

#### 5.1.1. UNM Dataset

We selected the UNM dataset for two reasons: (a) it provides systematic data of iterated program execution over a long duration, which is needed to learn all execution paths of a program, and (b) it is a well-known public dataset and has been used in recent IoT anomaly detection approaches, such as [41], which allows for a better comparative analysis. The dataset consists of normal and attack system traces for several programs, and we used the syscall traces of Sendmail and line print remote (LPR) programs. These are Unix utility programs used to route email and send print jobs to the printer. Comprehensive details of the dataset are provided in [56], and we briefly discuss the dataset in the context of the proposed approach.

**Figure 7:**
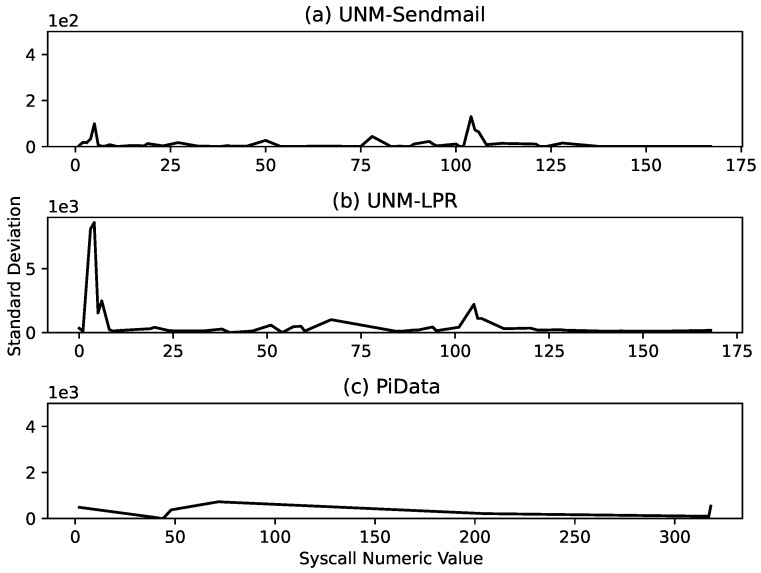
Dataset consistency analysis: Variation in frequency of syscalls of: (**a**) UNM Sendmail, (**b**) UNM-LPR, and (**c**) PiData datasets observed in 10 equal-sized segments. The plot shows the standard deviation in frequencies of three datasets’ syscalls. It can be seen that the frequencies of syscalls in the UNM Sendmail dataset do not vary much in different segments of the dataset, while syscalls in the UNM-LPR dataset show varied syscall frequencies.

A syscall trace is a long sequence of numeric values substituted against the original syscalls according to a mapping file. A program’s syscall trace contains syscalls from its main and child processes; for example, the Sendmail program’s syscall traces contain data from 25 processes overall. An initial study of the dataset revealed that some longer syscall traces contained multiple copies of smaller traces, and we discovered and removed these copies from the main trace. We also analyzed the consistency of the datasets by measuring the change in the frequency distribution of the system calls in different parts of the dataset. This was done by creating 10 equal segments of each dataset and computing the standard deviation of the frequency of each system call over 10 segments. The analysis results presented in Figure 7 indicate that the frequency of many system calls is inconsistent among the different segments of the UNM-LPR dataset. This analysis shows that the program behind the UNM-LPR syscall sequence has a relatively diverse execution behavior.

#### 5.1.2. Custom Dataset

The custom dataset, PiData, consists of five normal and four attack traces captured under different normal and attack conditions. All traces were captured from our custom IoT device built using a raspberry pi, a temperature & humidity sensor, and a remote broker (now called the server); both the client and server used the message queuing telemetry transport (MQTT) protocol [57] for communication, the details of which are provided in Table 3. The device application has two main modules: (a) an MQTT module for exchanging data with a remote server and (b) a sensing module to read data from a digital humidity and temperature sensor (DHT) DHT11.

##### Normal Execution Conditions

We maintained the following conditions for normal syscall data: (1) client-sending and server-only acknowledging, (2) client-sending while server-acknowledging and publishing periodically, and (3) similar to the previous case, with the exception that the server is publishing data randomly, for which we used another client that randomly published data on a server that consequently reached our IoT device. Five traces were collected under the aforementioned conditions and all five traces were used during training.

##### Attack Scenarios

For attack traces, we created an experimental setup for two attacks: (a) a code execution (CE) attack and (b) a packet drop attack. For the CE attack, we created an exploit condition and linked it with malicious code, which could be triggered by a remote client by publishing a specific message for our custom IoT device. Upon execution, the malicious code creates a reverse shell for a remote machine that uses it to execute shell commands on the devices.

**Figure 8:**
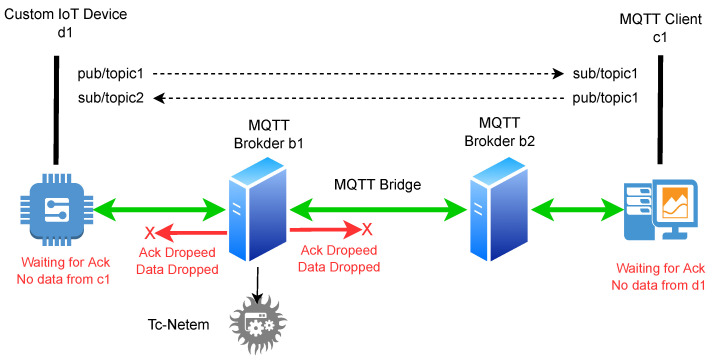
Laboratory setup for emulated denial of service (EDoS) attack on the custom IoT device. The black lines indicate the message settings, the green lines show normal MQTT traffic, and the red lines show the situations during the attack broker, b1, dropping traffic intended for d1 and c1.

Packet drop attacks [59], a type of denial of service (DoS) attack, were emulated in a laboratory environment, as shown in Figure 8. The setup consists of MQTT clients d1 and c1, where d1 is our custom device and two MQTT brokers, b1 and b2, are bridged together so that data published on b1 is delivered to all the devices connected to b2 and vice versa; the black and green lines show the normal communication prior to the attack, while red labels and arrows indicate the situation during the attack. We launched the attack on b1 by executing a traffic policy that drops 80% of the outgoing traffic, this is done by using a well-known Linux utility Netem [60]. An 80% drop was selected to keep the connections alive, as dropping all traffic would make the broker unavailable, and all connections were terminated. During this attack, data from our IoT device (d1) were dropped at b1 and were delivered to b2 and consequently to c1; however, the data from b1 did not reach d1, which caused long wait and resend queues at d1, and its normal operation was greatly disrupted. We captured several syscall traces during this attack, and selected the longest and shortest traces for diversity.

### 5.2. Evaluation Metrics

We first evaluated our proposed approach on the test data; from each dataset, we used 80% of the data for training and kept aside 20% for testing. We analyzed the performance using precision (9), recall (10), accuracy (8), and $F_{1}\;\mathrm{score}$ (11), for comparison with related work we also computed true and false positive rates (TPR,FPR). We provide a brief description of these performance metrics and their suitability for measuring the performance of the proposed approach.

(8) $$Accuracy=\frac{TP+TN}{TP+TN+FP+FN}$$

(9) $$Precision=\frac{TP}{TP+FP}$$

(10) $$Recall=\frac{TP}{TP+FN}$$

(11) $$F_{1}\;score=\frac{2*Precision*Recall}{Precision+Recall}=\frac{2*TP}{2*TP+FP+FN}$$

A syscall trace evaluated by our proposed approach can consist of all normal, all malicious, or a mix of both segments; a normal segment detected as normal is counted as true positive TP, otherwise considered as false negative FN, and a malicious segment detected as an anomaly or attack is counted as true negative TN otherwise taken as false positive FP. Equations (8)–(11) provide definitions of precision, recall, accuracy, and $F_{1}$ score, all of which measure the important characteristics of the model under evaluation. Precision is a good measure of the model’s false positive rate, and high precision indicates that the model is good at detecting normal as normal; however, it misses false negatives, i.e., how many times a normal segment is counted as an anomalous segment. Similarly, recall is a measure of false negatives that ignores the number of times an anomalous segment is detected as a normal segment. Both accuracy and $F_{1}$ score provide a holistic view of the system’s performance according to the cost of FN and FP; however, accuracy is more suitable for situations where the costs of FN and FP are the same, i.e., it is equally important or damaging to detect a normal segment as an attack or vice versa.

## 6. Results and Comparison

**Figure 9:**
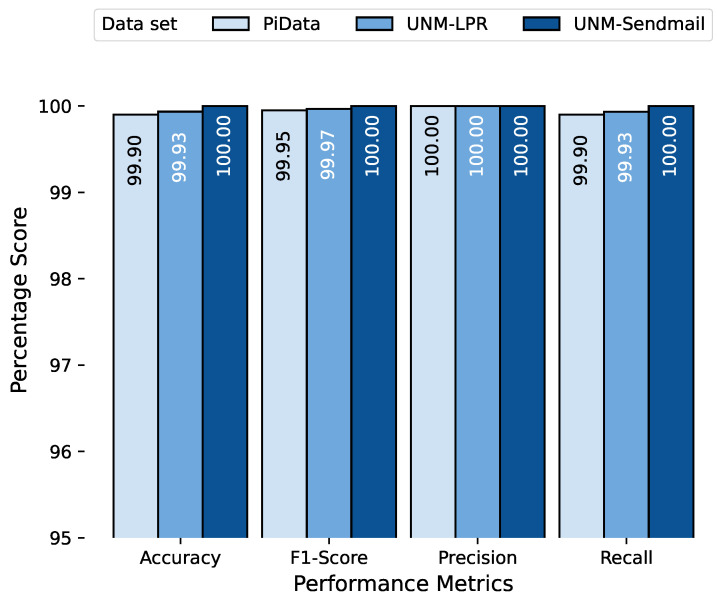
Results of the executing proposed anomaly detection approach over test data of all three datasets.

We first discuss the performance of our proposed approach on test data. The combined testing results of all datasets are shown in Figure 9, and the four performance metrics of each dataset are grouped together for better comparison. The results indicate that our proposed approach performed excellently over the test data with a perfect score (100%) on the UNM Sendmail dataset and near-perfect scores (99.9% and 99.9%) on PiData and UNM-LPR datasets, respectively. This difference between the performances is because the UNM Sendmail dataset is relatively cleaner and consistent compared with the other two datasets, as indicated in Figure 7.

**Figure 10:**
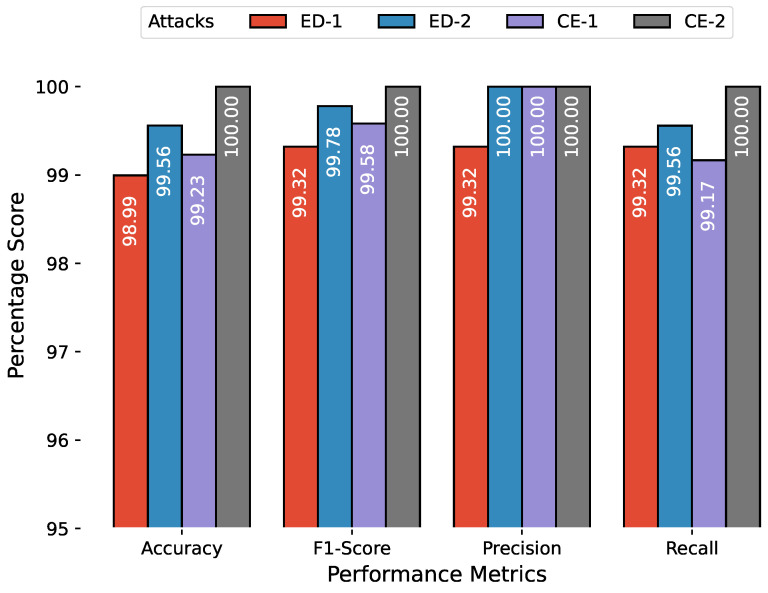
Anomaly detection results of PiData dataset, the attack abbreviations are; ED: emulated DoS, CE: code execution.

**Figure 11:**
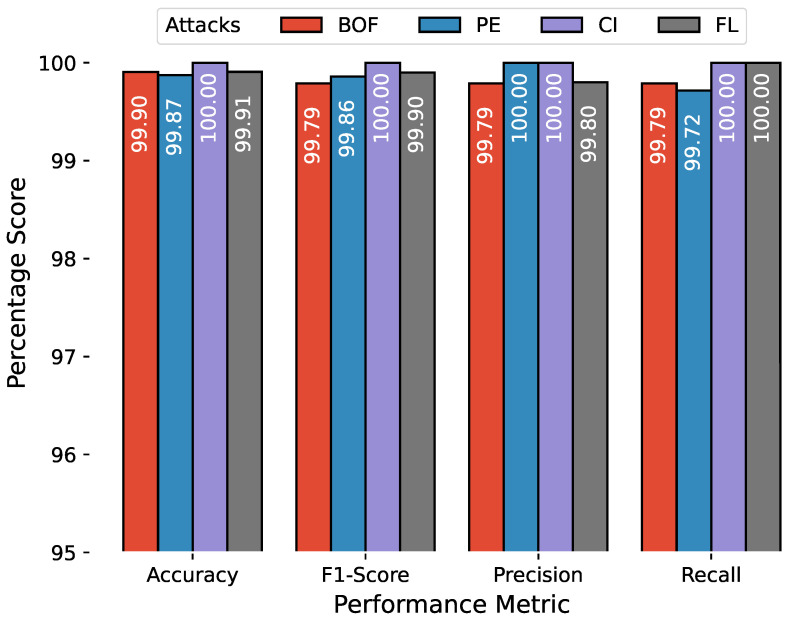
Anomaly detection results of UNM Sendmail dataset, the attack abbreviations are; BF: Buffer overflow, PE: Privilege Escalation, CI: Code Injection, FL: Forwarding Loop.

**Figure 12:**
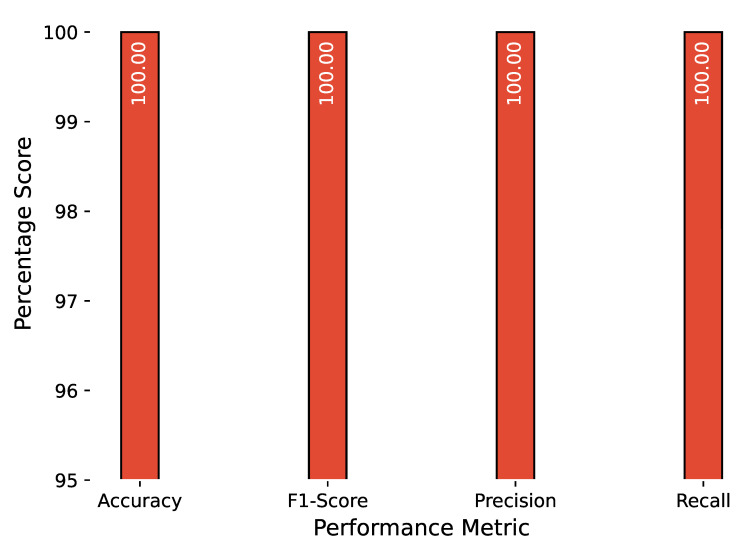
Anomaly detection results of UNM-LPR dataset.

The anomaly detection results are combined in a similar manner, and Figure 10, Figure 11 and Figure 12 show the accuracy, $F_{1}$ score, precision, and recall for attacks related to PiData and UNM Sendmail dataset attacks. The results of multiple attack traces from the same category have been averaged, for example, the results for four traces of buffer overflow attacks are individually computed and averaged.

For comparison, we selected closely related works published recently, Table 4 provides the comparison details. Each of the selected studies is closely related to the proposed approach. The approach suggested by Mirsky et al. [30], lightweight collaborative anomaly detection for the IoT using blockchain (CIoTA), has these commonalities with our approach (a) sequence-based anomaly detection (b) Markov chain for transition probabilities (c) target domain, i.e., IoT. Similarly, the approach proposed by Liao et al. [41], anomaly detection of system call sequence based on dynamic features and relaxed-SVM (ADSC-DFRS), has many similarities with our study: (a) both are system-based IoT anomaly detection approaches, (b) both use the UNM dataset for evaluation, and (c) both consider the segmentation of the syscall sequence. Finally, the approach introduced by Toan et al. [48], a novel approach to detect IoT malware by system calls and long short-term memory model (IMD-SLSTM), is based on syscalls sequence analysis and attempts to find the optimum syscall subsequence length.

Table 5 provides a performance comparison of the proposed approach with related works in terms of the reported performance metrics. The comparison table shows that the overall performance of the proposed approach is better than those of the other approaches considered for comparison. The minimum FPR of CIoTA matches the FPR of the proposed approach’s FPR, however, CIoTA has relatively low accuracy and a high maximum FPR value. IMD-SLSTM and ADSC-DFRS have accuracies that are relatively closer to the proposed approach’s accuracy however, they have low $F_{1}$ scores. IMD-SLSTM does not report FPR, whereas ADSC-DFRS has a relatively high FPR.

### 6.1. Computation Cost

To determine the computational performance of the proposed approach, we measured the utilization of the central processing unit (CPU), memory consumption, and execution duration under different conditions. The performance parameters were measured using Psrecord [61] and custom Python scripts. Psrecord is an open-source command line utility for monitoring and recording the memory and CPU-related metrics of a given program or process. We emphasized measuring the performance of the anomaly detection module of the proposed approach, as this module is specifically designed for IoT devices. The performance experiments were executed on two different devices, one of which is a custom IoT device and the other one is a laptop. The details of the custom IoT device are provided in Table 3, the laptop consists of a 120 GHz processor with four cores and 8 GB of memory. Data used during execution on both devices comprised a normal syscall trace consisting of 64,000 syscalls and 14 attack traces comprising approximately 13,000 syscalls taken from the UNM Sendmail test and attack dataset.

To analyze the execution performance of IoT devices, we deployed the trained anomaly detection model on our custom IoT device and executed it in two different modes: (a) as a script using an integrated development environment (IDE) and (b) as a standalone executable. In both cases, the device maintained its routine tasks, i.e., sensing the temperature and humidity, and sent it to the remote MQTT broker. Table 6 provides the percentage of CPU utilization, mean memory, and total time consumed by the anomaly detection module on both devices, i.e., custom IoT and laptop in both scenarios. During execution, 2006 and 410 syscall segments were detected by the anomaly detection module in the test and attack data, respectively. The last column of Table 6 shows the CPU time required to process a syscall segment of average length, which in this case is approximately 32 syscalls for both the test and attack syscall data. The execution of the proposed anomaly detection module finished much sooner on a laptop in all scenarios than on the custom IoT device because of the differences in the computation resources of the two devices. In both devices, the execution was much more efficient in the IDE because of the efficient handling of resources by the IDE such as loading libraries and managing memory.

Standalone executions turned out to be costly, as a standalone executable is embedded with the required libraries that it has to load and manage at the time of execution. The code that we deployed on both devices is of an experimental nature, which is neither optimized for efficiency nor implemented for any specific platform. The proposed anomaly detection approach uses simple arrays, matrices, and basic mathematical operations, which allow efficient implementation in low-level programming languages, such as C/C++.

### 6.2. Discussion

The reason for this high performance is that, for a syscall sequence to be normal, first, its length should closely fall into the length group derived from normal segments; second, it should have a probability greater than or equal to the minimum probability in its length group. This justifies our reason for segmenting using SoE, i.e., it allows us to determine the correct length of normal syscall sequences. The length of a given syscall sequence directly affects its probability, as each syscall sequence is considered a trajectory of the Markov chain, and the probability is a product of state transition probabilities in the trajectory, as shown in Equation (3). This justifies the role of Markov chains in the proposed anomaly detection scheme.

Next, we discuss the reasons for the high precision and recall scores. A syscall sequence can be anomalous for many reasons, including, but not limited to, (a) it has a length greater or smaller than the length of normal sequences, (b) it has syscalls not observed in normal syscall sequences, and (c) the order of syscalls is different from that exhibited by normal syscall sequences. We have already discussed the role of length for being normal or anomalous, unobserved syscalls lead to unknown state transitions, i.e., from observed to new syscall and from new syscall to observed syscall, an unknown state transition is the one that has no entry in the transition matrix, and its value is considered either zero or very low Section 3.2, as discussed in Section 3.2 the overall probability of such a syscall sequence will be way lower than the normal threshold. In the case of anomalous syscall sequences that do not contain any unseen syscall and whose lengths are closer to the lengths of normal syscall sequences, it is highly probable that the proposed approach considers them as normal syscall sequences. This issue is addressed by assigning weights to syscalls in a syscall sequence according to their position in the sequence. In the proposed approach, we use the position of syscalls in the syscall sequence as its weight.

After the training process, we evaluated the proposed approach using test and attack data. For each case, we measured the precision and recall of the number of segments that were processed and correctly identified as normal or malicious. For the test data, we did not need to actually verify how many segments were actually present in the test data, but to expect that none of the segments were identified as malicious as there were no malicious segments in the test data. Each attack trace has some part of the normal execution of the program, as each attack is executed after the program starts normally. For attack traces, the proposed method correctly identifies both the normal and malicious or anomalous parts of the traces. We verified the anomaly detection results for attack traces in two ways: (a) by manually identifying parts of the trace as normal and malicious and (b) by removing the normal part from the attack trace.

Although the method we propose to find the start of the execution pattern SoE is efficient, it requires training data that restricts this approach to learn directly from the device data in real time. A possible future direction is to develop an online approach that can learn the start of the execution of a target application directly from device data. Linked to this is the problem of acquiring syscall data directly from the device in an online manner. During this study, we also realized that acquiring syscall traces using external tools is tedious, which hinders the performance of the overall approach. An improvement to this shortcoming can be embedding a non-blocking syscall acquisition mechanism into the anomaly detection approach itself. This can be done in many ways, from developing custom syscall trace modules to using open-source utilities. In the present approach, we used numeric values of syscall only and ignored the call parameters. In the future, will investigate the possibility of using call parameters to learn normal execution behavior. One final thought for our future work is to avoid full deployment of the anomaly detection approach on the device itself to avoid deployment and device resource issues. We believe that the approach should be developed in such a way that only a minimal part is placed on the target device, such as the device data acquisition module, and the complex anomaly detection part should be kept in an external machine. Such an approach would be highly flexible and extensible as a change or update in an anomaly detection module would not require re-deployment on IoT devices.

## 7. Conclusions and Future Work

In this study, we propose a lightweight and efficient host-based anomaly detection approach for IoT devices. The proposed approach addresses two key limitations of the existing approaches in this area: a predetermined probability threshold and fixed-length segmentation. Addressing these two issues allows for better generalization of anomaly detection approaches in this domain. The proposed approach has been evaluated using a variety of datasets against several well-known attacks. The performance has been reported in terms of standard performance metrics and compared with recently published work. The proposed approach provides a novel and effective method for handling syscall data for anomaly detection in IoT devices. This study can serve as a basis for future syscall-based anomaly detection approaches. 

## Figures and Tables

**Table 1:** Summary of the related work that highlights the used approaches, methods, and datasets.

Article	Approach	Method	Dataset
Sivanathan et al. [26]	IoT network flow analysis	Model checking	Custom
Sivanathan et al. [27]	Network packet analysis	Classification, K-Means	Custom
Eskandari et al. [28]	Network flow analysis	Classification, LOF & IF	Custom
Maniriho et al. [29]	Network traffic analysis	Classification, RF	IoTID20
Mirsky et al. [30]	Memory jump sequence analysis	Markov Chain	Real Data
Nguyen et al. [31]	Federated Learning	Classification, GRU	Custom
Wang et al. [32]	Device usage rules analysis	Custom framework	From [47]
Sharma et al. [36]	Specification analysis	Formal Verification	None
Forrest et al. [37]	Syscall sequence analysis	Sequence matching	Custom
Toan et al. [48]	Syscall sequence analysis	Classification, LSTM	Custom
Liao et al. [41]	Syscall sequence analysis	Classification, SVM	UNM
Shobana et al. [42]	Syscall n-gram analysis	Classification, LSTM	Custom
Hoang et al. [40]	Syscall n-gram analysis	Classification, SVM	Custom
Liu, Z et al. [43]	Syscall statistical pattern analysis	IF, LOF, KNN, SVM	ADFA
Zhang, Y. et al [44]	Syscall behavioral semantics	Classification, TextCNN	ADFA-LD
Breitenbacher et al. [45]	Process whitelisting	Hash tables, SHA256	Real Devices
Carter, J et al. [46]	Feature engineering	Feature pruning, PCA	Custom

**Table 2:** Syscall datasets details: Custom-PiData is our laboratory-generated dataset, and UNM Sendmail and UNM-LPR are public datasets. Column normal data provides details of traces and the number of instructions in each dataset, the column attack provides names of the attacks and the number of traces for each attack.

Normal Data				Attack Data	
**Dataset**	**Traces**	**Instructions**		**Attack Description**	**Traces**
				Privilege Escalation	03
				UNM Sendmail-decode	02
				Forwarding Loops	05
PiData (Custom)	05	1,322,032		Emulated DoS	02
				Code Execution	02
UNM-LPR	4500	2,027,468		Code Injection	1000
MIT-LPR	2700	2,926,304		Code Injection	1000
Total 4	7211	7,847,387		7 different attacks	1018

**Table 3:** Characteristics of the IoT device.

Feature	Value
Raspberry Pi model	4B
Raspberry Pi memory	4 GB
Messaging protocol	MQTT 5.0
Temperature and humidity sensor	DHT11
Server/Broker	Eclipse Mosquitto [58]
Programming language	Python 3

**Table 4:** Comparison of characteristics of related anomaly detection approaches with the proposed approach.

Parameter	CIoTA [30]	IMD-SLSTM [48]	ADSC-DFRS [41]	Proposed
Domain	IoT	IoT	Generic	IoT
Anomaly Detection	Y	N	Y	Y
Syscall Based	N	Y	Y	Y
Dataset	Custom	Custom	UNM, ADFA-LD	Y
Threshold	Fixed	Dynamic	Dynamic	Dynamic
Segmentation	None	Fixed	Mixed	Dynamic
Sequence-Based	Y	Y	Y	Y
Markov chain	Y	N	N	Y

**Table 5:** Comparison of the performance with existing approaches, some performance metrics are not reported by all selected approaches and are therefore left blank.

Approach	Year	Accuracy		$F_{1}$ Score		FPR	
		Max	Min	Max	Min	Max	Min
CIoTA [30]	2019	96.9	91.60	-	-	2.14	0.0
IMD-SLSTM [48]	2021	98.37	-	98.38	-	-	-
ADSC-DFRS [41]	2022	99.00	-	93.00	-	2.40	-
Proposed	2022	100.00	99.87	100.00	99.86	0.86	0.0

**Table 6:** Performance analysis of the proposed approach.

Device	Data	Execution	CPU (%)	Memory	Time (s)	Total	Time/TS (s)
		Scenario		(MB)		Segments (TS)	
Custom IoT	Test	Standalone	28.43	37.86	16.91	2006	0.008
Custom IoT	Test	Using IDE	24.9	16.8	13.12	2006	0.006
Custom IoT	Attack	Standalone	22.25	20.5	2.23	410	0.005
Custom IoT	Attack	Using IDE	6.55	12.8	9.2	410	0.022
Laptop	Test	Standalone	31.46	14.8	0.98	2006	0.002
Laptop	Test	Using IDE	23.2	20.25	0.33	2006	0.0001
Laptop	Attack	Standalone	30.49	5.02	0.73	410	0.001
Laptop	Attack	Using IDE	1.45	17.2	0.23	410	0.0005

## Data Availability

Not applicable.

## Abbreviations and Notations

5G	fifth generation mobile network
ACF	autocorrelation function
CE	code execution
CNN	convolutional neural network
DHT	digital humidity and temperature
DoS	denial-of-service
DUR	device usage description
*f*	anomaly detection function
FN	false negative
FP	false positive
FPR	false positive rate
GRU	gated recurrent unit
IDE	integrated development environment
IF	isolation forest
Industry 4.0	fourth industrial revolution
IoT	Internet of Things
$l_{c}$	length of candidate segment
LCS	longest common subsequence
LPR	line printer remote protocol
LOF	local outlier factor
LSTM	long short-term memory
*M*	transition matrix
MC	Markov chain
MQTT	message queuing telemetry transport
MUD	manufacturer usage description
Netem	network emulation
OS	operating system
$p_{c}$	probability of candidate segment
*p*	transition probability
PiData	custom dataset
RF	random forest
$s_{c}$	candidate segment
SoE	start of execution
SVM	support vector machine
Syscall	system call
*T*	set of thresholds
TN	true negative
TP	true positive
TPR	true positive rate
UNM	The University of New Mexico
